# Towards Computational Modeling of Human Goal Recognition

**DOI:** 10.3389/frai.2021.737327

**Published:** 2022-01-19

**Authors:** Shify Treger, Gal A. Kaminka

**Affiliations:** ^1^ Gonda Multidisciplinary Brain Research Center, Bar Ilan University, Ramat Gan, Israel; ^2^ Computer Science Department, Bar Ilan University, Ramat Gan, Israel

**Keywords:** goal recognition, cognitive modeling, human recognition, intention recognition, plan recognition

## Abstract

Recently, we are seeing the emergence of plan- and goal-recognition algorithms which are based on the principle of *rationality*. These avoid the use of a plan library that compactly encodes all possible observable plans, and instead generate plans dynamically to match the observations. However, recent experiments by Berkovitz (Berkovitz, The effect of spatial cognition and context on robot movement legibility in human-robot collaboration, 2018) show that in many cases, humans seem to have reached quick (correct) decisions when observing motions which were far from rational (optimal), while optimal motions were slower to be recognized. Intrigued by these findings, we experimented with a variety of rationality-based recognition algorithms on the same data. The results clearly show that none of the algorithms reported in the literature accounts for human subject decisions, even in this simple task. This is our first contribution. We hypothesize that humans utilize plan-recognition in service of goal recognition, i.e., match observations to known plans, and use the set of recognized plans to conclude as to the likely goals. To test this hypothesis, a second contribution in this paper is the introduction of a novel offline recognition algorithm. While preliminary, the algorithm accounts for the results reported by Berkovitz significantly better than the existing algorithms. Moreover, the proposed algorithm marries rationality-based and plan-library based methods seamlessly.

## 1 Introduction

The last dozen years are seeing the emergence of plan- and goal-recognition algorithms which are based on the principle of *rationality* (Ramirez and Geffner, 2010; Sohrabi et al., 2016; Vered et al., 2016; Vered and Kaminka, 2017; Kaminka et al., 2018; Masters and Sardina, 2019). These avoid the use of a plan library that compactly encodes all possible observable plans, and instead generate plans dynamically, on-the-fly, to match the observations. They therefore offer an approach to eliminating one of the fundamental assumptions of most recognition methods of the field, since its inception in the late 1970s (Schmidt et al., 1978). Moreover, their reliance on rationality seems to be compatible with studies of human recognition capabilities (Baker et al., 2007; Baker et al., 2009; Bonchek-Dokow and Kaminka, 2014).

In a sense, these rationality-based recognition algorithms (henceforth, *RGR* for Rational Goal Recognition) do goal recognition first, plan recognition second. The likelihood of hypothesized goals is evaluated on the basis of the *dynamically-generated* plans, rather than pre-existing knowledge of potential plans. In particular, RGR algorithms dynamically generate optimal (i.e., rational) plans that match the observations as best as possible. These plans are compared against other generated plans, which are not constrained to match the observations^1^. The comparison between these plans leads to conclusions as to which goal is more likely, given the observations (see Section 2 below for detailed treatment).

Recently, we came across a series of experiments carried out by Berkovitz and Parush (reported in (Berkovitz, 2018)), which may cast some doubt as to the use of RGR as a model of human recognition capabilities. In particular, Berkovitz and Parush examined a relatively simple goal-recognition task, where a robot arm moves towards one of two possible goal locations. On different trials, the motion of the arm followed different trajectories: Sometimes, an optimal (direct) trajectory to a goal, and other times, a curving, inefficient trajectory. A cursory examination of their results shows that in many cases, humans seem to have reached quick (correct) decisions when observing motions which were far from optimal, while in other cases, incorrect (and much slower) decisions were reached when observing optimal motions. The results also showed the reverse; the difference did not appear to lie in the optimality of the trajectory.

Intrigued by these findings (which we detail in Section 3) we created a simulation of the experiments carried out in (Berkovitz, 2018), allowing us to evaluate various recognition algorithms on approximately the same data. We experimented with a variety of rationality-based state-of-the-art goal-recognition algorithms, contrasting their predictions with the data from the human subjects^2^. The results clearly show that none of the algorithms reported in the literature accounts for human subject decisions, even in this simple task. This is our first contribution.

We hypothesize that humans utilize plan-recognition in service of goal recognition, i.e., plan-recognition first, goal recognition second. We believe humans match observations to known plans, and use the (sometimes ambiguous) set of recognized plans to conclude as to the likely goals, in stark contrast to RGR algorithms. We conjecture that the within-subjects experiment design in (Berkovitz, 2018) primed the subjects to expect the type of inefficient trajectories they may observe. In other words, the subjects formed a plan library in the early parts of the experiment, which they utilized in latter stages.

To test this hypothesis, a second contribution in this paper is the introduction of a novel offline recognition algorithm, Library-based Rational Goal Recognition (LRGR). The proposed algorithm marries rationality-based and plan-library based methods. It can handle incomplete plan libraries (indeed, with no plan library, it is identical to rationality-based recognition methods), while still utilizing a plan library when available. While preliminary, the algorithm accounts for the human subject results much better than the existing algorithms.

We conducted additional experiments contrasting LRGR, RGR, and idealized (classic) library-based plan-recognition on plan recognition problems, varying the size of the plan library available. Experiments in close to 800 randomly-generated goal-recognition problems in the same domain demonstrate that LRGR consistently outperforms both RGR and the classic library-based plan recognition methods on this data set. Furthermore, LRGR seems remarkably robust to plan-library size and incompleteness with respect to the observations.

## 2 Background: Goal- and Plan- Recognition

Plan, Activity, and Intent (goal) Recognition (collectively called *PAIR*) is the field of study in AI which investigates how one agent, observing another, may infer on the basis of these observations the other agent’s plan of action, the goal of the plan, or the class of activity taking place. Generally, this is an abductive inference task: the observations are partial with respect to the full plan (the full sequence of actions); the goal is not observed, etc. In plan recognition the focus is mostly on recognizing the sequence of future actions. In goal (sometimes called intent) recognition we are interested in inferring the final goal state of the agent. A comprehensive survey of the field, which has started in the late 1970s (Schmidt et al., 1978), is well beyond the scope of this paper. We refer the interested party to several comprehensive recent surveys by Sukthankar, Goldman, Geib, Pynadath and Bui (Sukthankar et al., 2014), by Mirsky, Keren, and Geib (Mirsky et al., 2021), and by Van-Horenbeke and Peer (Van-Horenbeke and Peer, 2021), as well as to an earlier survey by Carberry (Carberry, 2001).

Since its early beginnings, many—if not most—approaches to goal and plan recognition utilized *plan libraries* (Carberry, 2001; Sukthankar et al., 2014; Mirsky et al., 2021). These compactly encode *all* potential plans the observed agent may be carrying out, in a form allowing a plan recognition algorithm to efficiently identify plans matching the observations. The recognition of plans is carried out by identifying the plans, within the library, matching the observations. Each plan in the library is associated with a goal, and thus any recognized plan also leads to the recognition of its associated goal, i.e., plan recognition first, leads to goal recognition second. In general, several plans may match the same set of observations; often, probabilistic ranking of the recognition hypotheses is carried out by the plan recognition algorithm.

A great variety of recognition techniques relying on such plan libraries have been investigated, e.g., (Kautz and Allen, 1986; Carberry, 1990; Charniak and Goldman, 1993; Goldman et al., 1999; Pynadath and Wellman, 2000; Bui, 2003; Avrahami-Zilberbrand and Kaminka, 2005; Pynadath and Marsella, 2005; Blaylock and Allen, 2006; Avrahami-Zilberbrand and Kaminka, 2007; Geib and Steedman, 2007; Sukthankar and Sycara, 2007; Geib and Goldman, 2009; Kabanza et al., 2010; Bisson et al., 2011; Blaylock and Allen, 2014; Wiseman and Shieber, 2014; Mirsky et al., 2016; Mirsky and Gal, 2016; Chakraborti et al., 2017; Mirsky et al., 2017). These are used in a variety of applications, often those involving human-machine interactions: natural language (Cohen et al., 1981; Carberry, 1990; Charniak and Goldman, 1993), assistant agents (Lesh et al., 1999; Geib and Goldman, 2001; Babaian et al., 2002; Yang, 2009), computer games (Fagan and Cunningham, 2003), and intelligent tutoring systems (Greer and Koehn, 1995; Gal et al., 2008; Amir and Gal, 2013).

However, the fundamental assumption of a complete plan library accounting for all possible plans and all possible goals has proven to be a key challenge in many applications. For example, in applications requiring reasoning about continuous motions such as those recognizing human motion plans and goals (Devaney and Ram, 1998; Masters and Sardina, 2019), human drawings (Vered and Kaminka, 2017), or various human-robot interactions (Dragan et al., 2013; Kelley et al., 2014). But also in other domains, where the number of possible plans and goals is insurmountable, despite the relative simplicity of the domain. For example, the famous planning *blocks world domain* has infinite many possible goals and plans, despite having a small number of discrete actions, taking place in a discrete world made of objects, essentially all of the same type.

More recent approaches eliminate the use of plan libraries, and rely instead on domain models, often specified in a formal planning domain description language. The domain models can be used in several ways.

Some techniques are used to generate data-structures or heuristic knowledge offline, ahead of the observation input and recognition process (e.g., (Hong, 2001; Martin et al., 2015; Pereira et al., 2016; Pereira and Meneguzzi, 2016)). This generated information can then be used to identify goals, but without necessarily recognizing plans (in the sense of recognizing a full sequence of actions based on the partial set of observations).

A different set of techniques, which we call here RGR (for Rational Goal Recognition) generally evaluate the likelihood of a hypothesized goal by generating and comparing the costs of two dynamically-generated plans:

• First, the *optimal* (i.e., rational) plan to reach the goal, while matching the observations (i.e., observed actions are part of the plan).
• Second, the optimal plan for the same goal that is either unconstrained to match the observations (Ramirez and Geffner, 2009; Sohrabi et al., 2016; Freedman and Zilberstein, 2017; Masters and Sardina, 2017; Shvo and McIlraith, 2017; Vered and Kaminka, 2017; Kaminka et al., 2018; Masters and Sardina, 2018; Masters and Sardina, 2019), or is constrained to *not* match the observations (Ramirez and Geffner, 2010).

Generally speaking, the closer the costs of these two plans, the more likely the goal; RGR algorithms prefer goals that result from plans that match the observations on one hand, and are optimal (rational) on the other. The assumption is that the observed agent is *rational*, thus it is more likely that it is taking an optimal plan towards the goal. Note that in most of these methods (other than (Vered and Kaminka, 2017; Kaminka et al., 2018)), the dynamically-generated plan itself is not important, and is not used as part of a plan recognition process.

The RGR approaches are considered to be a significant step towards removing the plan library assumption existing in most recognition works. The alternative assumption—rationality of the observed agent—had been thought to be compatible with both observed synthetic agents, as well as human recognition skills and biases. For instance, Bonchek-Dokow and Kaminka propose a model for *human* intention prediction (goal recognition) (Bonchek-Dokow and Kaminka, 2014), similar to earlier [e.g., (Ramirez and Geffner, 2009)] and later approaches [e.g., (Vered and Kaminka, 2017; Kaminka et al., 2018)]. In comparing the model to human behavior, they showed that when an observed motion was on an optimal (direct) path towards a goal location that the subjects might not have thought of, the subjects dynamically hypothesized the existence of a new goal. The rationality of the motion was overriding previous knowledge of potential goals. Somewhat similarly, Baker *et al* (Baker et al., 2007; Baker et al., 2009) propose a model for human goal recognition, based on Bayesian inverse planning using Markov Decision Problems, and again relying on rationality. While their initial model did not agree with results from human subjects, two updated models, which took into account dynamic goal changes and subgoals within the sequence of actions, did simulate human recognition behavior.

Rational movement also was used as a component in planning robot motions that would be *legible* to human users. Dragan *et al* (Dragan et al., 2013) distinguish between predictability and legibility of robot motion and propose a mathematical formulation for both properties. They define predictability to be the observer’s expectation and they assume that the human observer expects the robot to be a rational agent. Therefore, the more optimal the motion, the more predictable. Legibility, in their definition, measures how easily the observer infers the goal from the observations. Therefore, the formula for legibility essentially integrates the probability that is assigned to the actual goal during the motion, with more weight given to the earlier parts of the motion.

Vered *et al* (Vered et al., 2016) presented an algorithm for online goal recognition in continuous environments, and tested it on data from human experiments, in two different recognition domains. Results show that for some goals, humans’ decisions were significantly different from the algorithm results. Vered *et al* concluded that humans use additional knowledge that is not part of the rationality-based model, but left further investigation to future work. This paper attempts to begin this investigation, by examining another experiment in which RGR algorithms failed to account for human recognition results.

## 3 The Berkovitz Experiments

In a recent study (Berkovitz, 2018), Berkovitz and Parush investigated some aspects of human spatial cognition in human-robot collaboration, in particular focusing on goal recognition. The study had human subjects observing a robot arm facing two goals standing on the surface. The robot arm would begin moving in a fixed speed towards one of the goals, which are equidistant from the robot. Subjects were asked to quickly identify the target goal location for the movement, by pressing one of two keys on a computer keyboard. They were motivated to choose correctly, but as quickly as possible. No second guesses were allowed.

The experiment followed a within-subjects design. Every one of 30 subjects was exposed to the same set of 14 different trajectories of the robotic arm towards one of the two goals (7 for each goal). The trajectories differ in their curvature, ranging from a straight line—the shortest path to each goal—to a very curved trajectory, which is inefficient. The order of exposure to the different trajectories was randomized.

**FIGURE 1:**
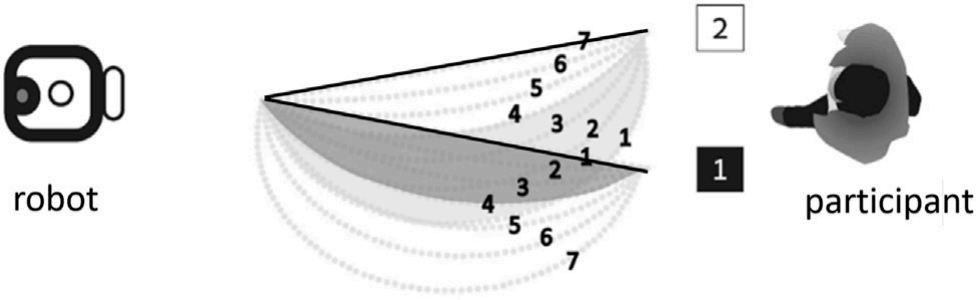
Berkovitz’s experiment trajectories 1–7 to goal 1, and trajectories 1–7 to goal 2. Trajectory 7 to goal 2, trajectory 1 to goal 1 (shown in bold) are optimal (*rational*). From (Berkovitz, 2018).

Figure 1 shows the experiment setup, viewed from above. This is referred to as the horizontal configuration, where the arm motions were on a horizontal plane. Trajectories were numbered according to their distance from the distractor goal (i.e., the other goal): For either goal, trajectory 1 is the closest trajectory to the other (distractor) goal, and trajectory 7 is the most distant from the other goal. The figure show some trajectories towards one goal overlap with trajectories to the distractor goal. For example, trajectory 4 to goal 1 overlaps initially with trajectory 2 to goal 2. The trajectories were not revealed to the subjects ahead of the experiment.

**FIGURE 2:**
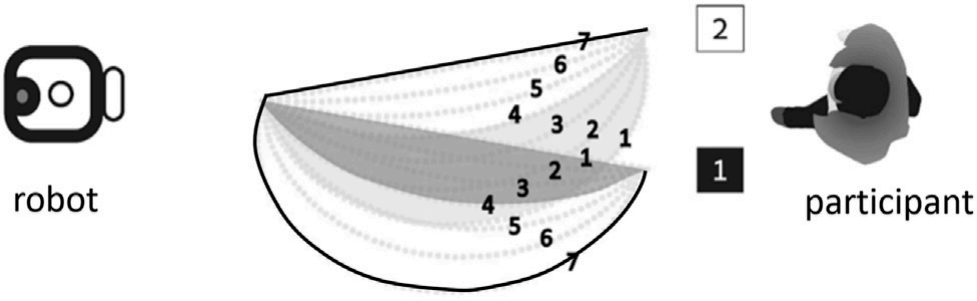
Trajectories denoted 7 (shown in bold) were *easiest* for the participants (faster, correct goal recognized). From (Berkovitz, 2018).

Analysis of the reaction time revealed that the distance of trajectories from the distractor was found to be a significant factor in their decision. Overall, participants tended to respond quicker to the trajectory that was most distant from the distractor (trajectories 7, for both goals). Figure 2 shows these trajectories, whose associated goals were recognized accurately and quickly. One of these trajectories is indeed optimal. The other one is the farthest from optimal as possible within the experiment design.

The results show that *the optimality of the observed trajectory was not the determining factor when humans inferred the correct goal*. People tend to answer quicker on trajectories 7 for both goals, but for one goal trajectory 7 is indeed the optimal trajectory while for the other goal it is not; indeed, it is not even close to rational.

Moreover, trajectories which can be seen to partially overlap (e.g., trajectory 4 to goal 1 overlaps initially with trajectory 2 to goal 2) caused the slowest responses from humans. Given that the trajectories were not revealed to the subjects ahead of time, how would the overlap (which to them would be unobserved) cause hesitation in committing to the recognized goal?

These results are not intuitive, considering the RGR approaches. The various RGR formulations generally evaluate the likelihood of a hypothesized goal by comparing the optimal plan that matches the observations, to the optimal plan for a goal (that is either unconstrained to match the observations, or even constrained to *not* match them). The RGR reasoning is that the more a plan that matches the observations is optimal (a rational choice of the observed agent), the greater the likelihood that the observed agent is attempting to reach the goal associated with the plan.

Intuitively, we would have expected the RGR algorithms to always choose the optimal plans (Figure 1). The human subjects instead preferred the most distant (Figure 2).

### 3.1 Goal Recognition Algorithms in Berkovitz’s Experiments

**FIGURE 3:**
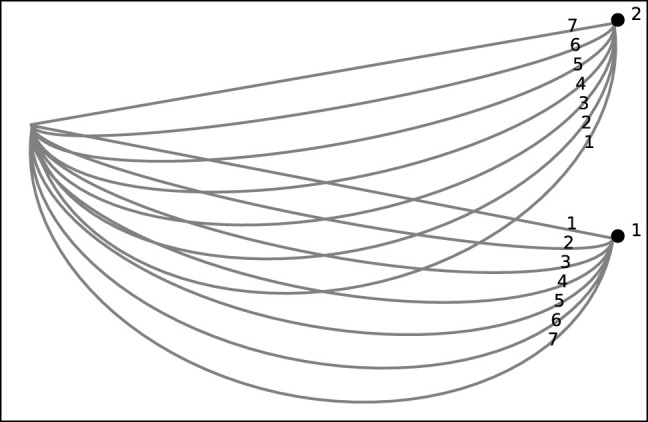
The trajectories in the simulation of Berkovitz’s experiments.

We attempt to go beyond intuition and qualitative assessment of the implications of Berkovitz’s experiments. To do this, we designed a 2D simulation that recreated Berkovitz’s horizontal configuration experiment (Berkovitz, 2018). It consists of an initial point and two goals. We designed 14 trajectories (shown in Figure 3), 7 towards each of the goals, from the initial point to one of the goals. Here, again, trajectories range from a straight, optimal trajectory to increasingly curved trajectories. We number trajectories similarly to Berkovitz’s study, from 1 to 7 indicating the distance from the distractor goal, beginning with 1 for closest to the distractor and 7 for the farthest from the distractor goal.

The simulation is built to allow experiments with various goal recognition algorithms, RGR or others. We generated observations for each of the 14 trajectories, in increasing length in relation to the trajectory’s length, simulating the motion of the arm along the trajectory in question. Each observation—increasing in length—is provided to each of the evaluated algorithms, and its responses are recorded. These responses are in the form of a probability distribution over the two goals.

We examined the following RGR algorithms on our simulation. In all (but one), the objective is to carry out goal recognition; computing *P* (*G*|*O*), i.e., the probability distribution of the goals in G where O is the set of observations. The exception is Dragan’s legibility algorithm, which ranks the goals based on the legibility of the trajectory observed.

The RGR algorithms evaluated in the simulation include:

• Ramirez and Geffner (Ramirez and Geffner, 2010). The algorithm computes

$$P\left(G|O\right)\propto e^{-\left(C\left(G,O\right)-C\left(G,\bar{O}\right)\right)}$$

where *C* (*G*, *O*) and $C\left(G,\bar{O}\right)$ are optimal costs for the respective plans for reaching the goals while matching the observations, and reaching the goals *without* matching the observations.

• Vered *et al* (Vered and Kaminka, 2017; Kaminka et al., 2018). The algorithm computes

$$P\left(G|O\right)\propto \frac{C\left(G\right)}{C\left(G,O\right)}$$

where *C*(*G*) is the cost of the optimal plan for a goal G and *C* (*G*, *O*) is the cost of the optimal plan that goes through the observations, as above.

• Masters and Sardina (Masters and Sardina, 2019). The algorithm computes

$$P\left(G|N,S\right)=\frac{e^{-\left(C\left(G,N\right)-C\left(G,S\right)\right)}}{1+e^{-\left(C\left(G,N\right)-C\left(G,S\right)\right)}}$$

where *N* represents the last observation in the observations set. (*C* (*G*, *N*) − *C* (*G*, *S*)) denotes the difference in costs between optimal plan from the last observation to a goal and the optimal plan from initial point to the goal. Their formula takes in account only the last observation.

• Dragan *et al* (Dragan et al., 2013) (*legibility*). The algorithm computes

$$legibility\left(\xi \right)=\frac{\int P\left(G*|\xi_{S\to \xi \left(t\right)}\right)f\left(t\right)dt}{\int f\left(t\right)dt}$$

integrating the probability of the actual goal along the trajectory with higher weight given to the earlier parts of the trajectory using a function like: *f*(*t*) = *T* − *t* with *T* the total length of the trajectory. To do this, it computes

$$P\left(G|O\right)\propto \frac{e^{-C\left(O\right)-C\left({\xi *}_{Q\to G}\right)}}{e^{-C\left({\xi *}_{S\to G}\right)}}$$

where *C*(*O*) is the cost of the trajectory going through the observations. *C* (*ξ*∗_*Q*→*G*_) and *C* (*ξ*∗_*S*→*G*_) are the costs of optimal trajectories from the last observation to the goal and from the initial point to the goal, respectively. This evaluates how efficient is going to a goal through the observed part of the trajectory in relation to the optimal trajectory. The idea is that more legible trajectories are easier to recognize, as they maximize the probability of the intended goal, in the mind of the observer.

**FIGURE 4:**
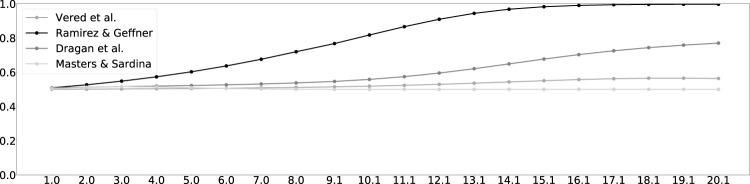
Trajectory 2 to goal 1. The horizontal axis measures the length of the observed trajectory. The vertical axis measures the probability of goal 1. The entire set of figures of the 14 trajectories can be found in Supplementary Appendix A.

Figure 4 shows the results of the algorithms, when observing simulated motion along trajectory 2 of goal 1. The horizontal axis denotes the length of the observed trajectory. The vertical axis denotes the likelihood of the associated goal, as estimated by the different RGR algorithms. The figure shows that while the algorithms differ in how quickly they recognize the goal (likelihood greater than 0.5), they all converge to the correct goal almost from the beginning of observing the motion. Similar results for all 14 trajectories are found in Supplementary Appendix A.

### 3.2 Comparing RGR and Human Recognition

A thorough consideration of RGR algorithms as a model of human recognition requires us to evaluate the fidelity of RGR algorithms to the human subject results. To do this, we examined the decision time of the simulated algorithms, which is conceptually equivalent to the measured human recognition response time (which measures the time of correct decisions by humans).

The decision-convergence (henceforth, *convergence*) time is the observed trajectory point for which both of the following conditions hold: 1) it is the *first* observed point in which the probability of the correct goal was greater than 0.5; and 2) no later observation had the value fall to 0.5 or below. In other words, it is the earliest point in the incrementally observed trajectory in which the algorithm recognized and committed to the correct goal. It is directly comparable to the mean normalized response time for correct decisions by the participants in Berkovitz’s study, which we include in Supplementary Appendix C.

**FIGURE 5:**
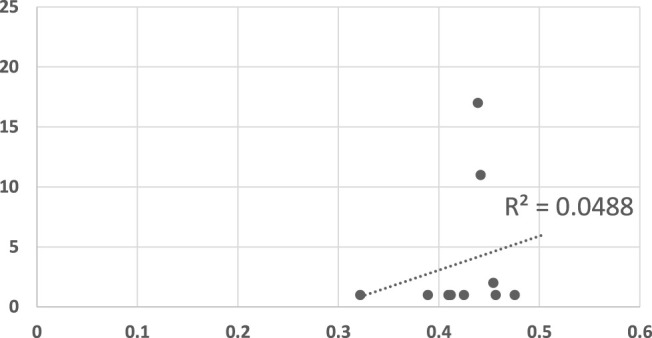
A scatter plot of human subject response times plotted against the convergence results of Dragan algorithm. The horizontal axis measures the human decision point as a fraction of the maximal trajectory motion duration. The vertical axis measures the algorithm decision point in terms of the observed trajectory length. The results are insignificant at *α* = 0.05, *p* = 0.533736143.

**FIGURE 6:**
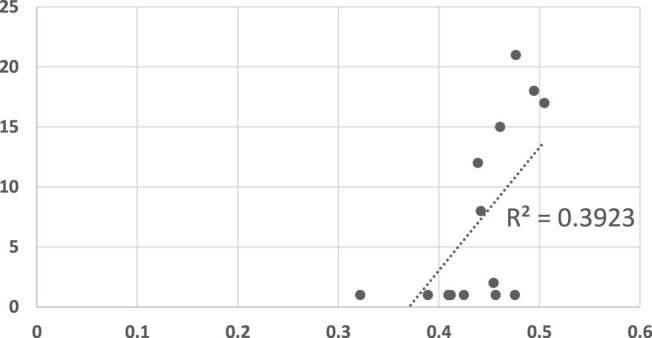
A scatter plot of human subject response times plotted against the convergence results of three RGR algorithm (the convergence results were identical for all three). The horizontal axis measures the human decision point as a fraction of the maximal trajectory motion duration. The vertical axis measures the algorithm decision point in terms of the observed trajectory length. The results are significant at *α* = 0.05, *p* = 0.016547283.

We examined the predictive power of the various algorithms by plotting the converge point of the algorithms against the mean response time of human participants in Figures 5, 6. In both figures, the horizontal axis measures the human response times in seconds, normalized to the maximum duration of motion. The vertical axis measures the observation point in which the algorithm converged (decided) on the correct goal.

Figure 5 shows the results generated by Dragan’s legibility algorithm (Dragan et al., 2013). A recognition algorithm that can perform well as a model for human responses would show as a plot where there exists a clear linear dependency between the two sets of responses. Clearly, Figure 5 shows no such visible tendency. The *R* ^2^ measure for this data is 0.0488, and the null hypothesis (no dependency exists), cannot be ruled out (*p* = 0.534, tested using the standard F-test for *R* ^2^). We note that the legibility score was intended to identify trajectories that are intended to be understood by the observer, while the motions which were observed by the human participants did not have this goal in mind. While some may be legible by themselves, legibility was not, overall, a good predictor of human response times in this experiment.

Figure 6 shows the results of the algorithms of Ramirez and Geffner (Ramirez and Geffner, 2010), Vered *et al* (Vered and Kaminka, 2017; Kaminka et al., 2018), and Masters and Sardina (Masters and Sardina, 2019). All three algorithms had identical convergence results, and are therefore plotted here together in the same figure (i.e., each of the 14 points was identical for all three algorithms). The three RGR algorithms are somewhat better as models of human recognition. The *R* ^2^ measure is 0.3923, and may be considered significant at *α* = 0.05 (*p* = 0.016547283).

**FIGURE 7:**
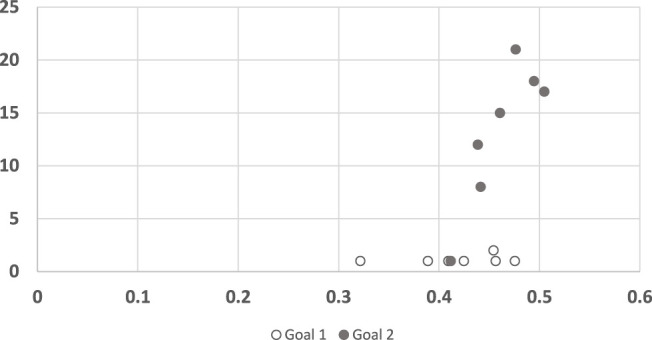
A scatter plot of human subject response times plotted against the convergence results of three RGR algorithm (the convergence results were identical for all three), clustered by goals. The horizontal axis measures the human decision point as a fraction of the maximal trajectory motion duration. The vertical axis measures the algorithm decision point in terms of the observed trajectory length.

Visibly, however, Figure 6 appears to have two clusters of points, only one of which might indicate a linear dependency. Indeed, upon further analysis (shown in Figure 7), it is clear that any dependency that may exist, is only towards Goal 2. For Goal 1, the algorithm convergence times are always low (quick decisions), while human responses vary (*R* ^2^ = 0.0902). In other words, the convergence points are unrelated to the trajectory, and instead depend on the goal.

**FIGURE 8:**
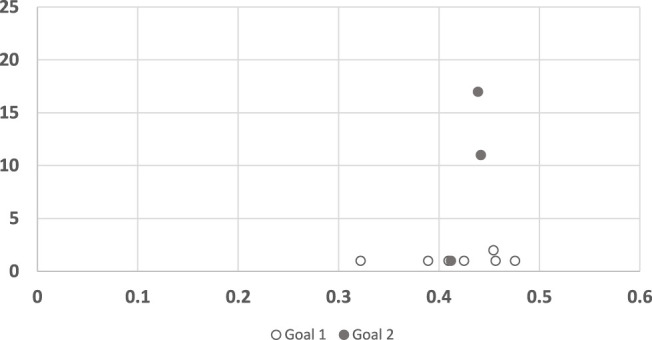
A scatter plot of human subject response times plotted against the convergence results of Dragan algorithm, clustered by goals. The horizontal axis measures the human decision point as a fraction of the maximal trajectory motion duration. The vertical axis measures the algorithm decision point in terms of the observed trajectory length.

For completeness, we also conducted the same analysis for the legibility algorithm (Figure 8). Here again, the results of the algorithm for trajectories to goal 1 seem consistently low (quicker convergence), with no correlation to human response time. For goal 2 the algorithm did not converge in 4 of the trajectories. In the other 3 trajectories it behaves remarkably different than in goal 1.

## 4 Recognizing Goals by Recognizing Plans

The results from the human subjects are not explained by RGR algorithms. The human subjects hesitate more, and make more mistakes, when observing trajectories that overlap with others. Given that the subjects only observe one motion trajectory at a time, why would they be confused by trajectories that are not observed?

Clearly, the subjects were affected by the virtual presence of overlapping trajectories (i.e., a plan library). But how would such a library form? Certainly, viewing a robot arm reaching for either of two goals was not part of the subject’s prior experience.

We conjecture that the *within-subjects* experiment design *primed* the subjects to expect the type of inefficient trajectories they may observe. In other words, we believe the subjects formed a plan library in the early parts of the experiment, which they utilized in latter stages. All subjects were exposed to all 14 incrementally-observed trajectories in random order. Only two of the trials would present optimal trajectories. The randomized order would result in the exposure of inefficient (curving) trajectories to all subjects within the first three trials, and likely, within the first one or two.

On the other hand, if we assume that the subjects only utilized their incrementally-formed plan library to carry out the recognition, then surely they would be more and more hesitant as more trajectories are observed, *regardless of the order of presentation*. The randomized presentation order, a standard practice in within-subjects experiment design, seeks to eliminate such ordering effects, and its use in this case indeed reveals that the hesitation is tied to the overlap between trajectories, not to the order of their presentation. Clearly, as evident in previous work discussed earlier (Section 2), humans are able to recognize goals even when the plans for them are novel to the observer.

We hypothesize that in stark contrast to both RGR and classic library-based plan recognition methods, humans mix both rationality-based and library-based recognition. We believe they match observations to the closest known plans (if such exist), and use the (sometimes ambiguous) set of recognized plans to conclude as to the likely goals. Failing to find a close plan, we believe they fall back to assuming the observed agent is rational, and follow an RGR-like procedure for recognition. In other words, we believe that while RGR algorithms *test each goal*, by checking the rationality of the dynamically-generated plan indicated by observations, humans *test each plan* for matching the observations, and only then check which goal(s) they lead to.

Specifically, we believe that when participants needed to perform goal recognition, observing a single trajectory, they utilized their primed, recent memories of similar trajectories. Rather than asking themselves which goal best matches an optimal plan, they focused on asking themselves what known plan was most similar or closest to what they are observing. Therefore trajectories that have more intersections with other trajectories to other goals will be more confusing, and so people tend to answer faster to trajectories that have fewer intersections with other trajectories toward another goal.

This section presents a recognition mechanism which embodies this hypothesis, and evaluates it on the original data and in comparison with RGR. The results show that the correlation with human subject response times is highly significant. As the mechanism relies on both rationality and plan library assumptions, the next section (Section 5) will also contrast its performance with both RGR and idealized library-based methods when the plan library is incomplete.

### 4.1 Library-Based Rational Goal Recognition Algorithm

LRGR (Algorithm 1) embodies a recognition method combining both rationality and plan library assumptions, in a preliminary form. It receives as input a set of goals *G*, a plan library *T*_*G*_, and an observation *o*. The plan library *T*_*G*_ is a set, partitioned into multiple sets *T*_*g*_, such that each subset *T*_*g*_ ⊂ *T*_*G*_ is associated with a goal *g* ∈ *G*. It then computes *P* (*G*|*O*) as follows.

First, LRGR iterates over all goals *g* ∈ *G*. For each one it examines every one of its associated trajectories *t* ∈ *T*_*g*_. Let us use *cost* (*p*1, *p*2) to denote the cost of the optimal path between two points *p*1, *p*2, and slightly abuse the notation, to also use *cost*(*t*) to refer to the cost of a trajectory *t* (even if it is not optimal). We use *last*(*t*) as the notation for the last point on some trajectory *t*, and *closest* (*p*, *t*) to denote the point on trajectory *t*, which is closest to the point *p*.

LRGR computes a hypothetical matching score *M*(*t*) for each trajectory *t* leading to each goal *g* (Lines 1–7). It considers the following conditions for such *t*.

• If the observed trajectory *o* directly matches the specific plan *t*, LRGR sets *M*(*t*) to 1 (Line 4).
• Otherwise, it computes an ad-hoc trajectory which matches the observations, but then goes to the closest point in the known trajectory *t* from the plan library, and continues optimally from there. This is compared to the cost of the optimal plan from the initial point to the goal.
*M*(*t*) in this case is set to the ratio between $cost\left(\hat{t_{g}}\right)$ (the cost of the optimal plan to the goal), and the cost *C* of the ad-hoc trajectory described above. In particular, *C* is the sum of:
• The cost of the observed trajectory (*cost*(*o*)).
• The cost of the path from the last point of the trajectory *o* to the closest point on the trajectory *t* (*cost* (*last*(*o*), *closest* (*last*(*o*), *t*))). If no close trajectory exists *closest* (*last*(*o*), *t*) is simply *last*(*o*) (the closest plan is the one observed), and thus the cost is 0 (*cost* (*last*(*o*), *last*(*o*)) = 0). This can happen if *T*_*g*_ is empty, or if *closest* () enforces a maximum distance cutoff.
• The cost of the optimal plan from that closest point on *t*, to the goal *g* (*cost* (*closest* (*last*(*o*), *t*), *g*)) (Lines 6–7, *C* is used here).
In line 8, LRGR chooses the maximum matching score *M* among the trajectories for each goal, because we are interested in a goal’s score and not in a trajectory’s score. Finally, we turn the matching scores to goal likelihoods by using the normalizing factor *η* = 1/(*∑*_*g*∈*G*_ *score*(*g*))

Algorithm 1 Library-based Rational Goal Recognition Algorithm (LRGR) (G, TG, o)
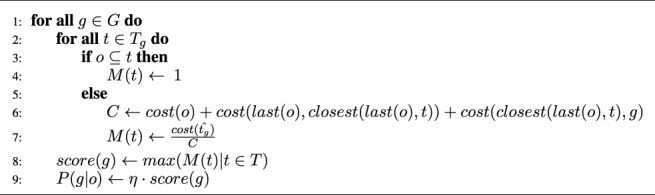

If an observation is a part of different trajectories to different goals, or is equally close to trajectories reaching different goals, then all such goals will get higher scores and thus the normalized probability scores for both goals will be lower, representing a more confusing observation. Similarly, if an observation is a part of only one trajectory to one goal, only this goal will get higher score and thus the probability normalized score will be higher score only for this goal.

The reader should note that the optimal plans for reaching the goals ${\hat{t}}_{g\in G}$ are not necessarily in the plan library. This does not preclude rationality-based recognition. In general, RGR algorithms evaluate rationality by comparing the observations against an optimal plan, to evaluate *P* (*G*|*O*). LRGR does the same: if the observations do not perfectly match a known plan (line 5), it constructs a hypothesized plan out of the observations (line 6), and compares its cost *C* to the optimal plan $\hat{t_{g}}$ . The comparison evaluates the rationality of the hypothesized plan.

Indeed, LRGR is closely related to *mirroring*, the RGR algorithm described in Vered et al. (Kaminka et al., 2018) (also tested in Section 3 above). In the special case where no closest plan can be found for a goal g the LRGR computation is identical to that of mirroring. Where mirroring has

$$P\left(g\in G|o\right)\propto \frac{cost\left(\hat{t_{g}}\right)}{cost\left(o\right)+cost\left(o,g\right)}$$

LRGR has

$$P\left(g\in G|o\right)\propto \frac{cost\left(\hat{t_{g}}\right)}{cost\left(o\right)+0+cost\left(o,g\right)}=\frac{cost\left(\hat{t_{g}}\right)}{cost\left(o\right)+cost\left(o,g\right)}$$

because when no close plan is found, the closest plan is the observed plan itself. However, because LRGR considers trajectories in the plan library for other goals, it may produce slightly different results when normalizing over *G*.

Computationally, LRGR may take significant resources, depending on the plan library representation and the planning processes used. It calls a planner to generate an optimal plan $\hat{t_{g}}$ for each goal, and also to generate a path from the last observation point to the closest point on a known trajectory. It makes a third call to the planner, to generate an optimal plan from that point to the goal.

### 4.2 Evaluation of LRGR on the Experiment Data

**FIGURE 9:**
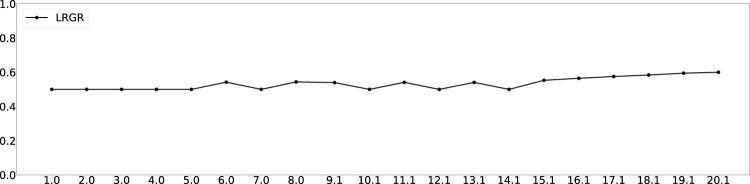
LRGR results on trajectory 2 to goal 1. The horizontal axis measures the length of the observed trajectory. The vertical axis measures the probability of goal 1. The entire set of figures of the 14 trajectories can be found in Supplementary Appendix B.

We followed the same experiment method described above, to evaluate LRGR on the same data as RGR. Figure 9 shows the results of the LRGR algorithm, when observing trajectory 2 of goal 1, analogously to the results of using the RGR algorithms on the same trajectory, as shown in Figure 4 in the previous section. Here again, the horizontal axis denotes the length of the observed trajectory and the vertical axis denotes the likelihood of the associated goal. Figures showing LRGR results for all trajectories are provided in Supplementary Appendix B.

The figure shows that the LRGR algorithm continues to change its estimate of the likelihood of the correct goal, from 0.5 and up and then back to 0.5 and so forth. The likelihood changes as observations match more than a single trajectory in the plan library. It therefore converges relatively later than the RGR algorithms (compare to Figure 4).

Qualitatively, this is novel behavior compared to the RGR algorithms, which differed in their likelihood estimation, but all converged identically quickly (in the case of this particular trajectory). Encouraged by this novelty, we next evaluate LRGR’s success in predicting human recognition response times. We follow the same setup as in Figures 5, 6.

**FIGURE 10:**
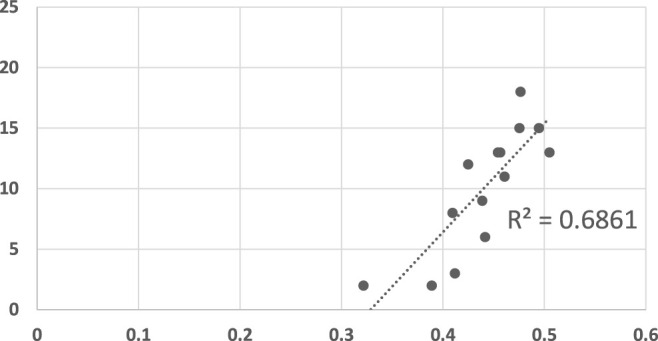
A scatter plot of human subject response times plotted against the convergence results of LRGR algorithm. The horizontal axis measures the human decision point as a fraction of the maximal trajectory motion duration. The vertical axis measures the algorithm decision point in terms of the observed trajectory length. The results are significant at *α* = 0.001, *p* = 0.00025244.

Figure 10 compares LRGR’s results to human reaction time. The axis are labeled identically to those of Figures 5, 6. The results visibly indicate a higher correlation (*R* ^2^ = 0.69), in comparison to the RGR results (*R* ^2^ = 0.39, maximally). Moreover, they are significant, even at a level of *α* = 0.001 (*p* = 0.00025244).

**FIGURE 11:**
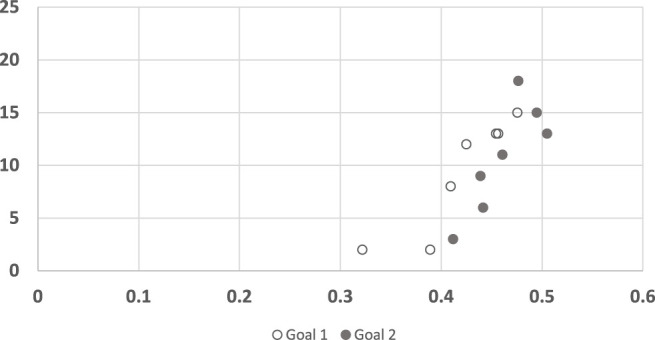
A scatter plot of human subject response times plotted against the convergence results of LRGR algorithm, clustered by goals. The horizontal axis measures the human decision point as a fraction of the maximal trajectory motion duration. The vertical axis measures the algorithm decision point in terms of the observed trajectory length.

Figure 11 shows the convergence results of LRGR, clustered by goals. Here, in contrast to RGR (see Figures 7, 8), the points of both goals are clearly correlated with human responses. The distribution of the data points is not influenced by the goals, but by the trajectories.

**FIGURE 12:**
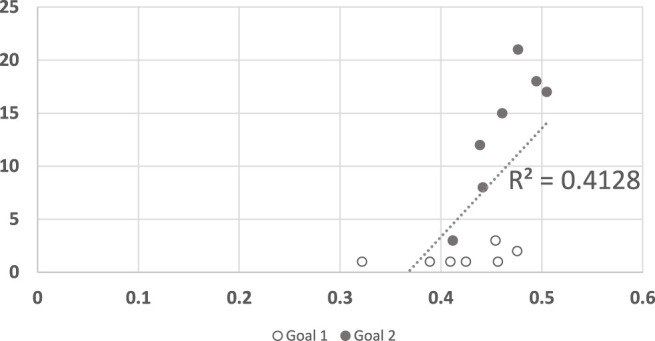
A scatter plot of human subject response times plotted against the convergence results of LRGR when utilizing optimal plans alone. The points are clustered by goals. The horizontal axis measures the human decision point as a fraction of the maximal trajectory motion duration. The vertical axis measures the algorithm decision point in terms of the observed trajectory length. The results are significant at *α* = 0.05, *p* = 0.035296632.

This is also a good opportunity to once again show that LRGR approximates RGR methods as the size of the plan library shrinks to zero. Figure 12 shows the results when LRGR relies on optimal plans alone. The results (and the *R* ^2^ value) are very close to those reported for RGR in the previous section (Figure 6).

## 5 Recognizing Plans With Incomplete Plan Library

The experiments carried out with LRGR in the previous section focus on LRGR versus RGR algorithms as models of human recognition processes, in the presence of a plan library. We provided the evaluated algorithms with the full library of plans which is going to be observed. The experiments therefore can only serve as evidence that human recognition relies (also) on a plan library, rather than rationality alone.

However, in such experiments, any library-based plan recognition algorithm would likely do equally well. Library-based plan recognition methods assume the plan library is complete^3^, and this assumption was satisfied in the experiments carried out in the previous section. RGR methods are intended to shine when the plan library is wholly missing; and this case was not evaluated in the previous section.

In this section we therefore explore the other assumption underlying LRGR’s operation: its reliance on a plan library. LRGR marries approaches assuming rationality or plan-library completeness, switching assumptions as needed. It uses the plan library as part of the dynamically-generated hypothesized plan, and then uses rationality to rank it. It therefore benefits where a plan library exists, but is does not assume it is complete.

A different set of experiments is needed to evaluate LRGR with respect to plan library incompleteness. We want to demonstrate that LRGR is able to handle observations of a trajectory, not in its plan library. Its performance should be contrasted with RGR methods (which are immune to changes in the plan library, for the simple reason they ignore it), and also to library-based methods which make the completeness assumption.

We compare LRGR to an idealized plan recognition algorithm, termed *Ideal* for the purposes of the discussion. The ideal plan recognition algorithm returns likelihood 1 if the observations match a known trajectory leading to a single goal, returns 0.5 if the observations match trajectories leading to 2 goals, and returns 0 if the observations match no known trajectory. As observations in this experiment come from trajectories which have overlaps, the results are not always 0.

The three sets of algorithms (RGR, LRGR, Ideal) are evaluated on goal recognition problems generated from the full set of 14 trajectories. We vary the size of the plan library given to the algorithms. The observed plan is *never* included in the plan library, yet the Ideal algorithm may still return a non-zero likelihood for a goal, as observations (from the unknown trajectory) may overlap with a trajectory that is included in the plan library.

We begin with the simplest set of generate goal recognition problems. In each such problem, the plan library includes only 13 trajectories from the set of 14 trajectories, and observations are generated from the 14th trajectory not included in the plan library. This is a “leave one out” design: as there are 14 trajectories in the full library, there are 14 problems generated by always leaving one trajectory out of the library and using it to generate observations.

While the “leave one out” design is simple, it has the benefit of being *realistic*, in the sense that it has certainly taken place in the experiments reported in (Berkovitz, 2018). As all 30 subjects were exposed to all 14 trajectories, they all encountered a recognition problem where 13 of the trajectories were already exposed to them, and they had to reach a decision upon observing a new one (which they have not previously observed).

**FIGURE 13:**
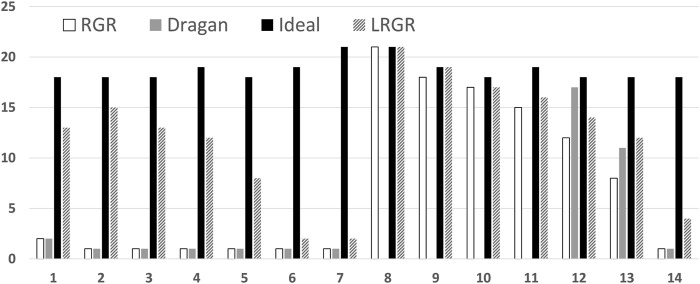
A comparison of the convergence results of different goal recognition algorithms. The horizontal axis marks the trajectory number left out. The vertical axis marks the convergence. Lower results indicate faster commitment to the correct goal.

Figure 13 shows the convergence time of each of the algorithms in each of the problems. The horizontal axis marks the trajectory left out. The vertical measures convergence, as before (quicker commitment, lower convergence point).

The figure shows that LRGR algorithms converge faster than other algorithms. These algorithms behave in the same way for observations that are part of the plan library and for new observations that are not included in the library, because they do not rely on any plan library. We remind the reader that our goal here is not to determine which algorithm is more accurate, or faster-to-commit, but instead to explore algorithms that model human goal recognition. As we have seen previously, the RGR algorithms do not necessarily provide a good model of human responses (e.g., for goal 1 trajectories).

Between the two algorithms utilizing a library (LRGR and Ideal), LRGR Algorithm converged faster than the ideal plan recognition algorithm. It is able to handle observations that do not match a plan, unlike the idealized plan recognition algorithm based on a plan library. It was therefore able to infer the correct goal based on similarity of the observations to a known plan.

We carried out another comparison (using the leave-one-out design), where we contrasted LRGR with the human data (which averages over every one of the participants, each one having been exposed to a leave-one-out recognition problem at the end of their experiment participation, as discussed above).

**FIGURE 14:**
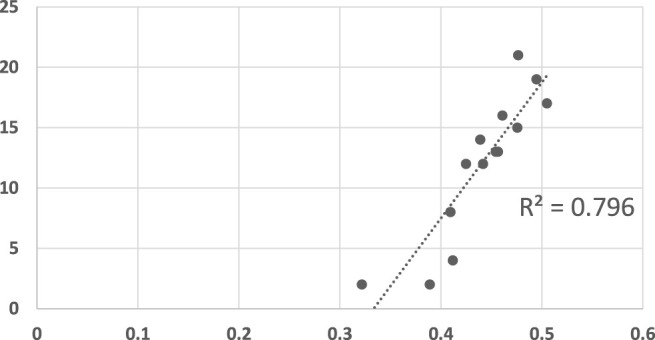
A scatter plot of human subject response times plotted against the convergence results of LRGR algorithm. The horizontal axis measures the human decision point as a fraction of the maximal trajectory motion duration (Supplementary Appendix C). The vertical axis measures the algorithm decision point in terms of the observed trajectory length. The results are significant at *α* = 0.0001, *p* = 0.00001789.

Figure 14 shows the results. The LRGR (Algorithm 1) convergence results are highly correlated with human recognition response times (*R* ^2^ = 0.796), *much* better than RGR algorithms. In fact, the results are significant at even higher level of significance (*α* = 0.0001, *p* = 0.00001789).

Encouraged by these results, we continued to generate recognition problems, where the size of the plan library was reduced. Each of the 14 trajectories was left out as an observation. Then, for each such trajectory, we generated a plan library by randomly choosing the needed number of trajectories (e.g., 3 trajectories for plan library of size 3) from among the 13 remaining in the original set (while guaranteeing that both goals were represented). We then evaluated LRGR’s convergence using the plan library. Put differently, given a controlled plan library size, we randomly generated 14 recognition problem. This was repeated 20 times for each plan library size (i.e., a total of 280 problems for each plan library size).

**FIGURE 15:**
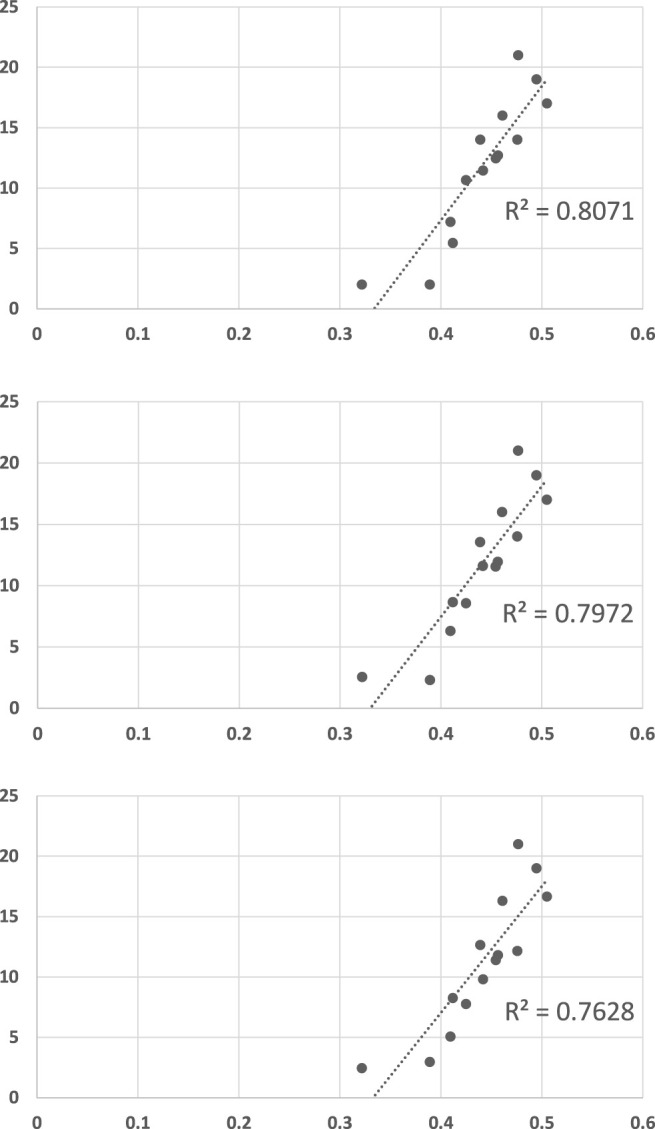
LRGR convergence versus human response times provided in Supplementary Appendix C, under conditions of different plan library sizes. Top to bottom: Plan libraries of size 10, size 6, and size 3 (compare to Figure 14, where the plan library size is 13). Each graph shows the results from 280 randomly-generated goal recognition problems. Each point is the mean of 20 trials.

Figure 15 shows the same type of scatter-plot graphs (and report on *R* ^2^ values) when the plan library size is restricted to 10 trajectories (top figure), 6 trajectories (middle figure), and 3 trajectories (bottom figure). Each such graph reports the mean of 20 recognition problems, for each of the 14 trajectories used as observations, i.e., a total of 280 goal recognition problems with a plan library of the given size. All *R* ^2^ values are significant at the *α* = 0.0001 level.

We believe that this last set of results provides strong evidence for the hypothesis that LRGR is able to function well with a (very) incomplete plan library, while able to also account for cases where the plan library has an important role in the recognition process.

## 6 Conclusion

We propose a novel approach to modeling human goal recognition mechanisms. Intrigued by Berkovitz and Parush’s study of human goal recognition, as reported in (Berkovitz, 2018), we hypothesized that humans recognize plans first (preferring known plans), and only on the basis of such recognition, infer (recognize) goals. This is in contrast with modern rationality-based goal recognition methods, where the likelihood of each goal is evaluated based on the rationality of a dynamically-generated plan matching the observations.

To test our hypothesis, we constructed a simulated version of the human subject experiment, and used it to evaluate known rationality-based goal recognition (RGR) algorithms. We found that RGR algorithms fail to account for human response times and recognition decisions.

We then develop a preliminary plan-library based goal recognition algorithm, called LRGR. The new algorithm uses plans in its library when they perfectly match observations. When they do not, it dynamically generates new plans accounting for the observations, and continuing through close known plans (i.e., close plans that *are* in the library). It therefore marries RGR and library-based plan recognition methods, taking the best of both approaches.

The comparison of mean reaction time of human subjects to the LRGR reveals that it is much better at predicting human recognition times (*R* ^2^ = 0.69 instead of *R* ^2^ = 0.39). In addition, we simulated scenarios in which the plan library is incomplete, allowing us to investigate LRGR versus idealized plan recognition algorithms that rely on a plan library, with no dynamic generation of plans matching the observations. We found that regarding accuracy of predictions, RGR algorithms work better than plan recognition algorithms. We also found that the new LRGR algorithm has better performance than an ideal plan recognition. However, RGR algorithms fail to model human recognition results. Lastly, we therefore compared the algorithms’ performance on new observations with human reaction time. Here, LRGR matches human behaviour even more clearly than earlier experiments (*R* ^2^ = 0.796). Further testing on approximately 800 randomly-generated recognition problems with different plan library sizes shows LRGR is remarkably robust to plan library incompleteness.

We consider this work to be only a first milestone towards a new approach for modeling human goal and plan recognition processes. While we are excited about this promising direction, we caution that the success reported in this paper is limited to a single experiment domain. While this domain has been specifically constructed to shed light on human recognition processes (Berkovitz, 2018), LRGR has not been yet tested against human response data in a different task. Also, as a model of human recognition process, it suffers from the same limitations as many RGR methods (from which it drew inspiration): For example, it is unable to recognize hierarchical goals and goal schema (Lesh and Etzioni, 1995; Blaylock and Allen, 2006; Blaylock and Allen, 2014).

We believe LRGR raises many opportunities for the readers interested in plan recognition in synthetic agents. We plan to continue development of LRGR and evaluate its modeling efficacy in different human tasks. We also plan to examine the computational implications of LRGR, in comparison to both RGR methods and library-based methods.

## Data Availability

The original contributions presented in the study are included in the article/Supplementary Material, further inquiries can be directed to the corresponding author.

## Footnotes

1 Depending on the actual algorithm used, they may even be constrained to *not* match the observations.

2 We gratefully acknowledge the help of Avi Parush and Inbal Berkovitz in understanding the experiments, and for providing us with the necessary data for comparison. See also Supplementary Appendix C.

3 Observations are assumed to perfectly match at least one plan in the plan library
