# Supplementary material for: Towards Computational Modeling of Human Goal Recognition
Source: Front Artif Intell. 2022 Jan 19;4:737327. doi: 10.3389/frai.2021.737327 (PMC8834086; doi:10.3389/frai.2021.737327)
Supplement: Supplementary file 1 [file DataSheet1.pdf]

APPENDIX A. RGR ALGORITHM RESULTS ON EACH TRAJECTORY

This appendix shows the results from running the RGR algorithms on all 14 trajectories.

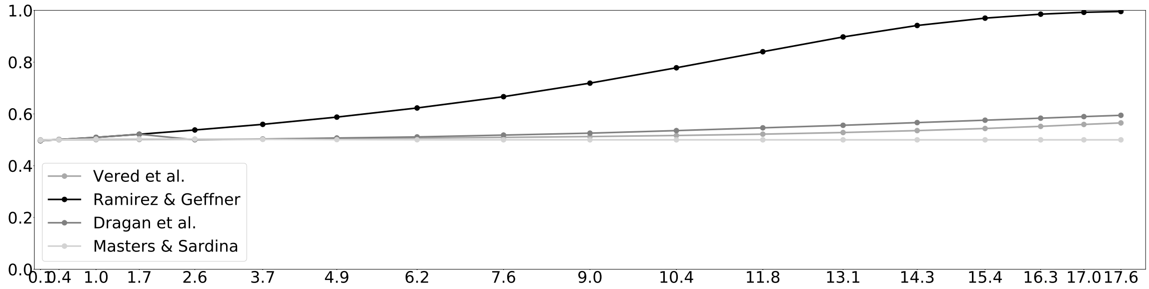

Figure 16. Trajectory 1 to goal 1. The horizontal axis measures the length of the observed trajectory. The vertical axis measures the probability of goal 1.

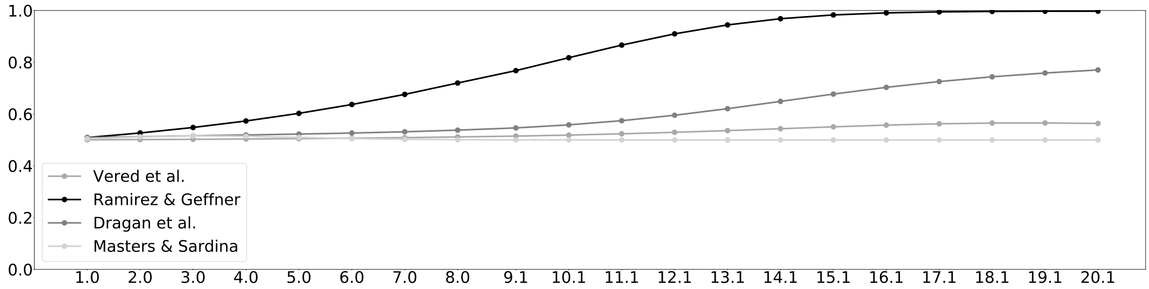

Figure 17. Trajectory 2 to goal 1. The horizontal axis measures the length of the observed trajectory. The vertical axis measures the probability of goal 1.

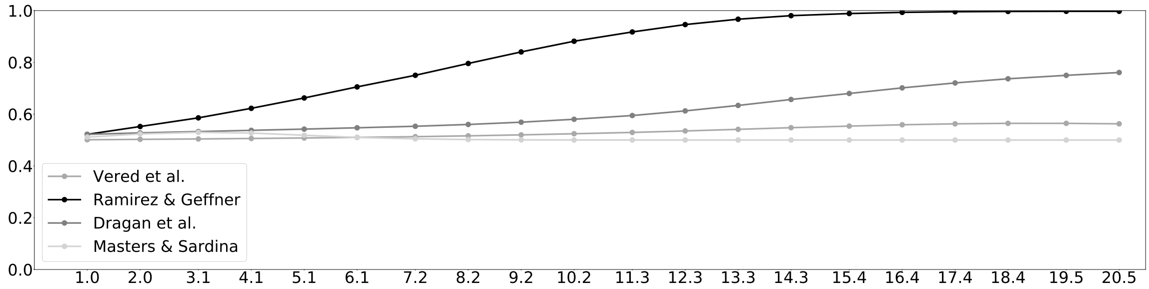

Figure 18. Trajectory 3 to goal 1. The horizontal axis measures the length of the observed trajectory. The vertical axis measures the probability of goal 1.

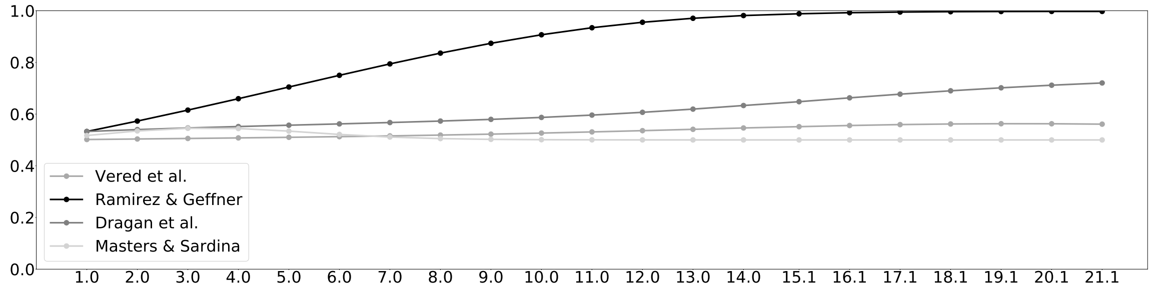

Figure 19. Trajectory 4 to goal 1. The horizontal axis measures the length of the observed trajectory. The vertical axis measures the probability of goal 1.

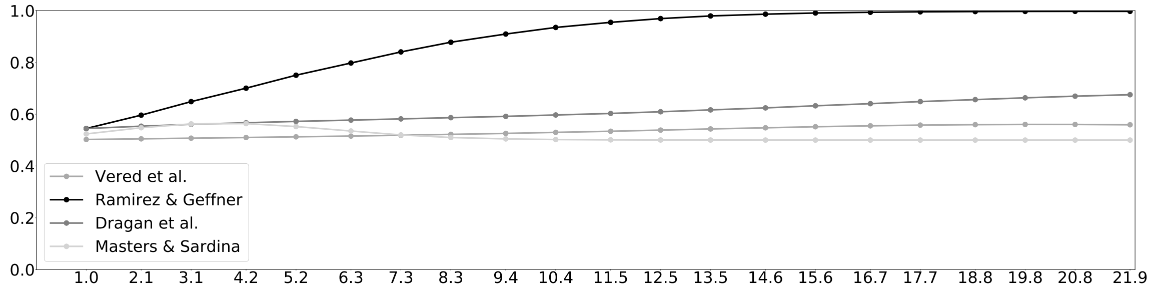

Figure 20. Trajectory 5 to goal 1. The horizontal axis measures the length of the observed trajectory. The vertical axis measures the probability of goal 1.

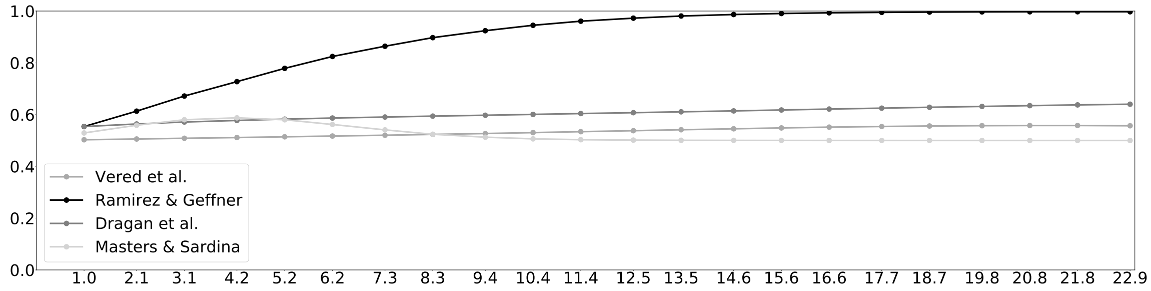

Figure 21. Trajectory 6 to goal 1. The horizontal axis measures the length of the observed trajectory. The vertical axis measures the probability of goal 1.

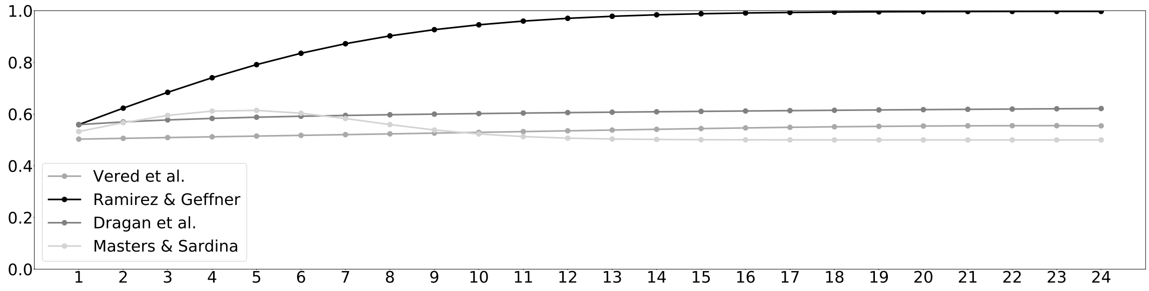

Figure 22. Trajectory 7 to goal 1. The horizontal axis measures the length of the observed trajectory. The vertical axis measures the probability of goal 1.

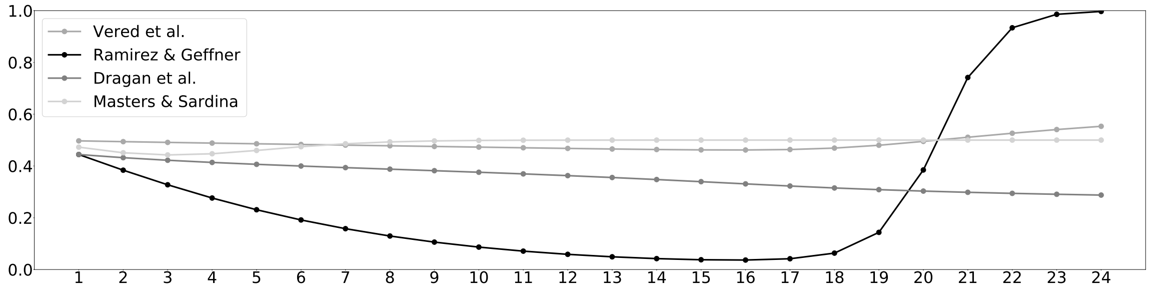

Figure 23. Trajectory 1 to goal 2. The horizontal axis measures the length of the observed trajectory. The vertical axis measures the probability of goal 2.

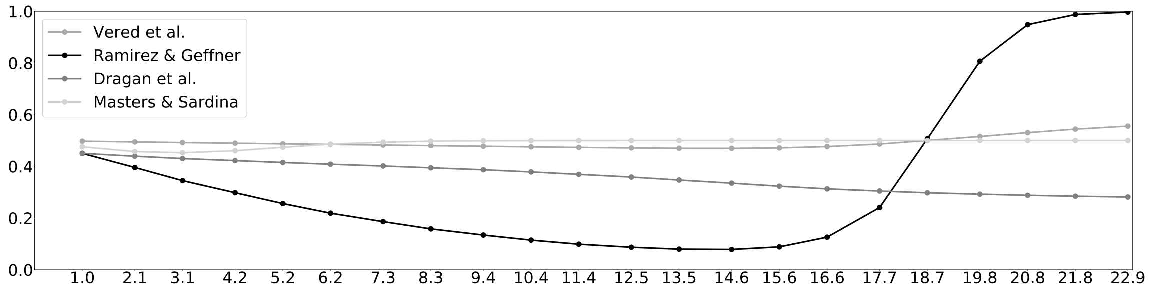

Figure 24. Trajectory 2 to goal 2. The horizontal axis measures the length of the observed trajectory. The vertical axis measures the probability of goal 2.

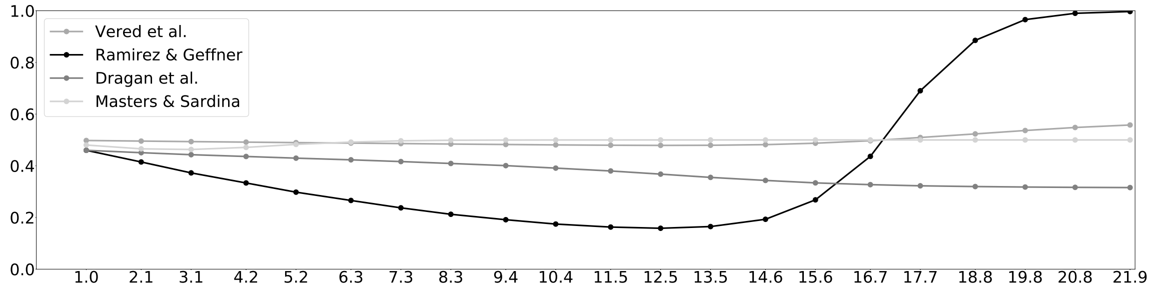

Figure 25. Trajectory 3 to goal 2. The horizontal axis measures the length of the observed trajectory. The vertical axis measures the probability of goal 2.

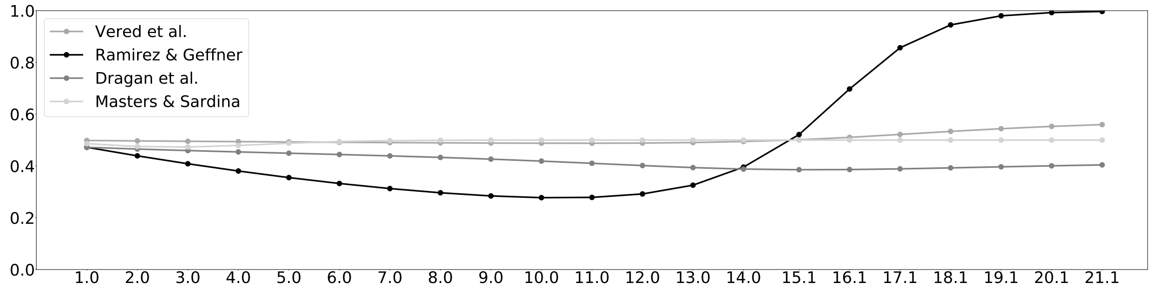

Figure 26. Trajectory 4 to goal 2. The horizontal axis measures the length of the observed trajectory. The vertical axis measures the probability of goal 2.

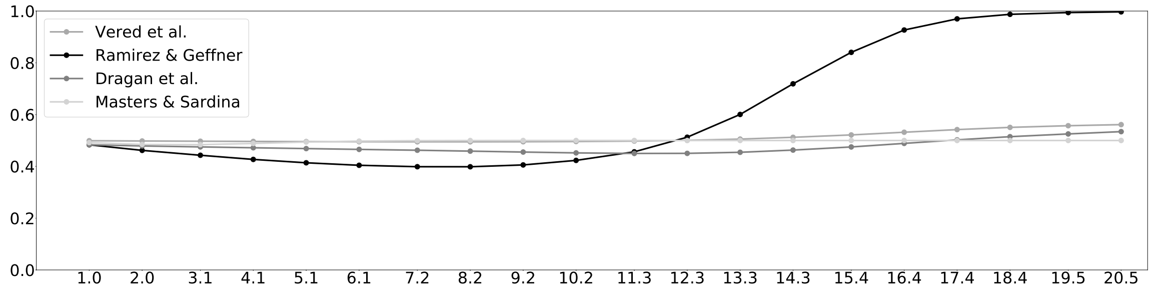

Figure 27. Trajectory 5 to goal 2. The horizontal axis measures the length of the observed trajectory. The vertical axis measures the probability of goal 2.

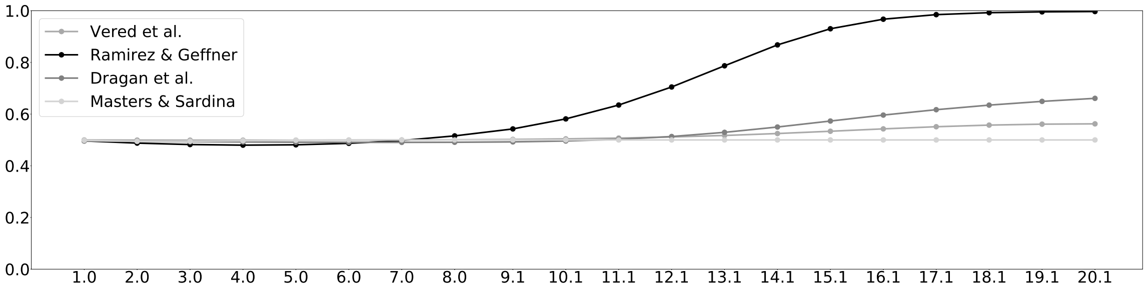

Figure 28. Trajectory 6 to goal 2. The horizontal axis measures the length of the observed trajectory. The vertical axis measures the probability of goal 2.

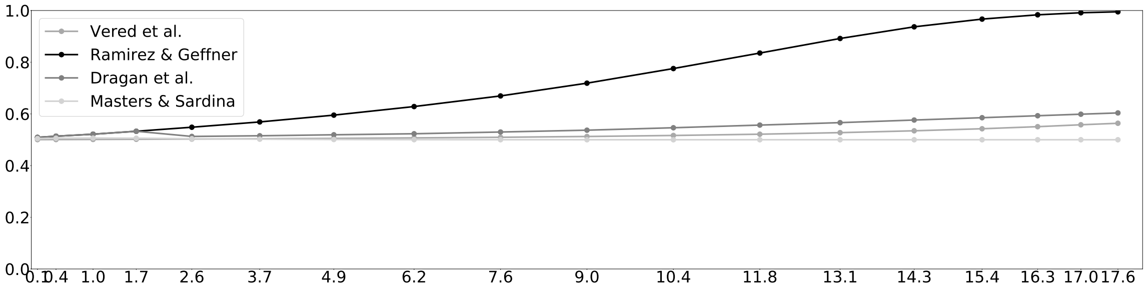

Figure 29. Trajectory 7 to goal 2. The horizontal axis measures the length of the observed trajectory. The vertical axis measures the probability of goal 2.

APPENDIX B. LRGR ALGORITHM RESULTS ON EACH TRAJECTORY

This appendix shows the results from running LRGR on all 14 trajectories. Figures are ordered identically to the order of figures in Appendix A, for comparison.

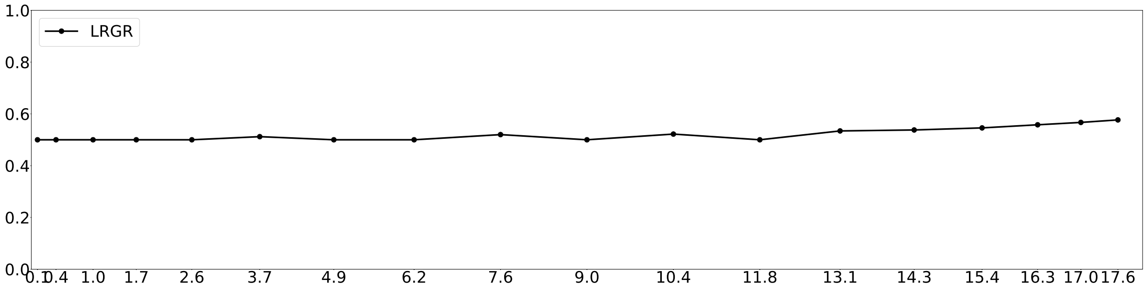

Figure 30. Trajectory 1 to goal 1. The horizontal axis measures the length of the observed trajectory. The vertical axis measures the probability of goal 1.

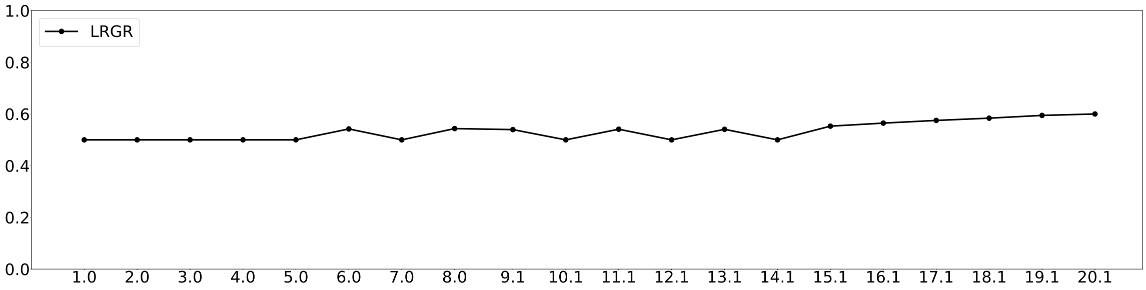

Figure 31. Trajectory 2 to goal 1. The horizontal axis measures the length of the observed trajectory. The vertical axis measures the probability of goal 1.

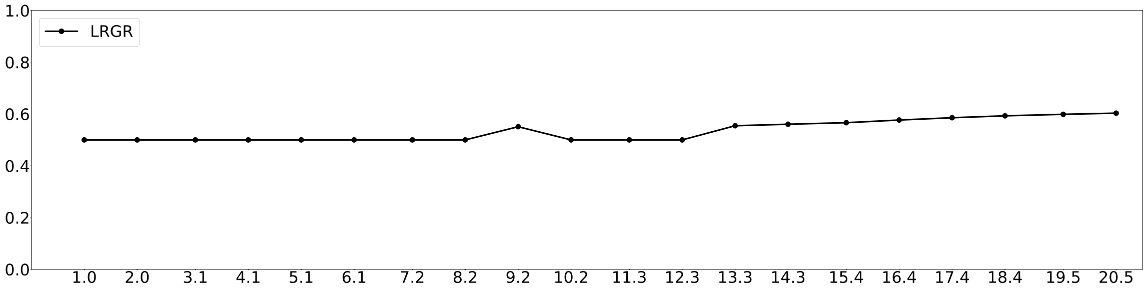

Figure 32. Trajectory 3 to goal 1. The horizontal axis measures the length of the observed trajectory. The vertical axis measures the probability of goal 1.

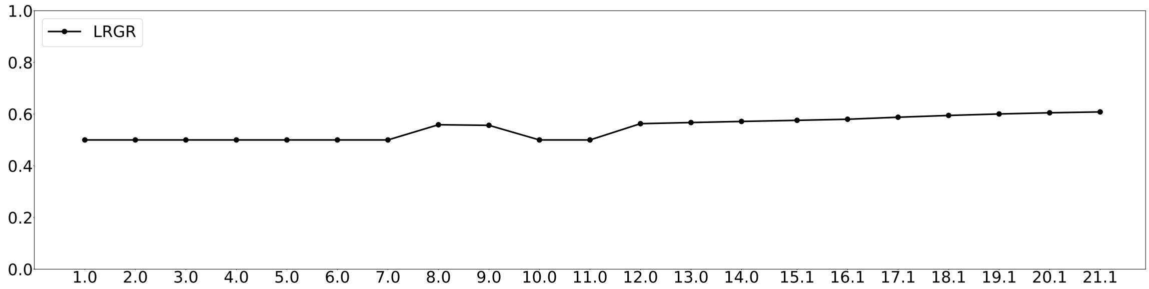

Figure 33. Trajectory 4 to goal 1. The horizontal axis measures the length of the observed trajectory. The vertical axis measures the probability of goal 1.

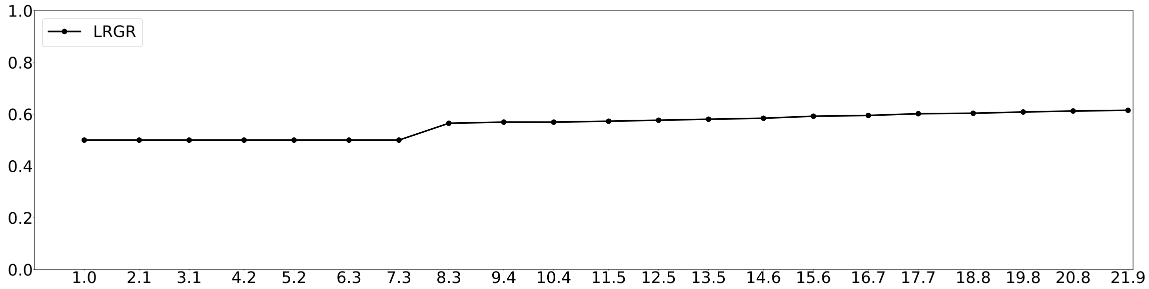

Figure 34. Trajectory 5 to goal 1. The horizontal axis measures the length of the observed trajectory. The vertical axis measures the probability of goal 1.

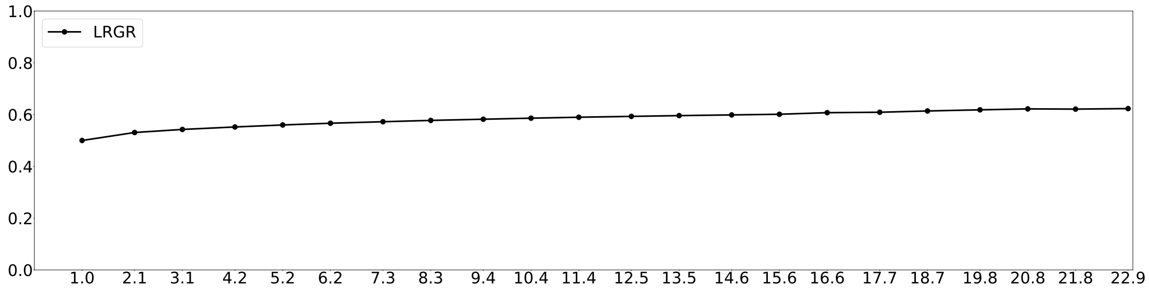

Figure 35. Trajectory 6 to goal 1. The horizontal axis measures the length of the observed trajectory. The vertical axis measures the probability of goal 1.

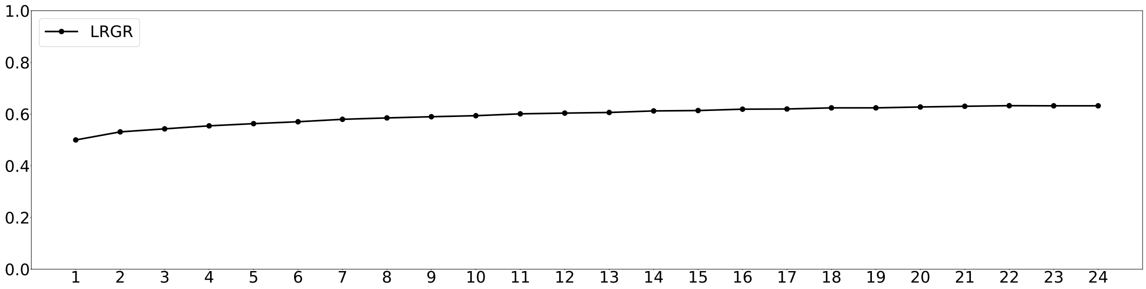

Figure 36. Trajectory 7 to goal 1. The horizontal axis measures the length of the observed trajectory. The vertical axis measures the probability of goal 1.

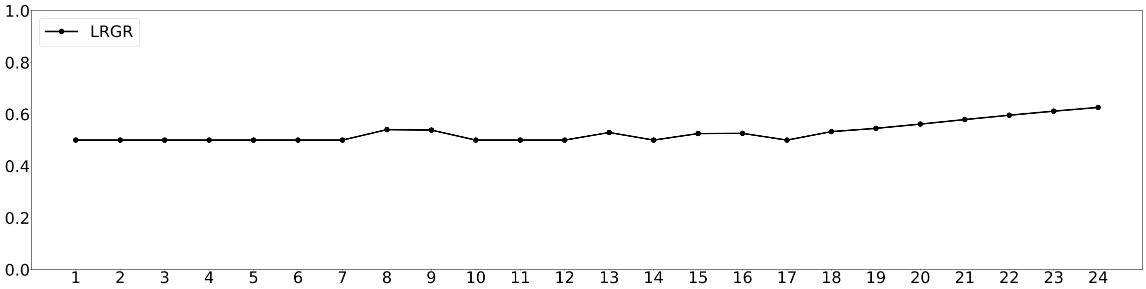

Figure 37. Trajectory 1 to goal 2. The horizontal axis measures the length of the observed trajectory. The vertical axis measures the probability of goal 2.

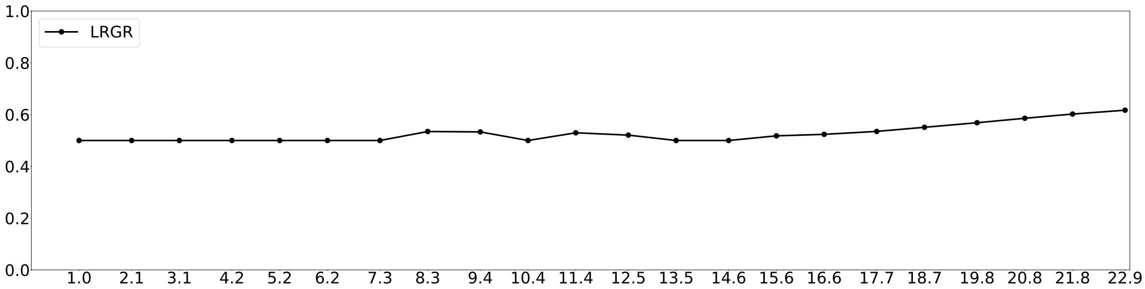

Figure 38. Trajectory 2 to goal 2. The horizontal axis measures the length of the observed trajectory. The vertical axis measures the probability of goal 2.

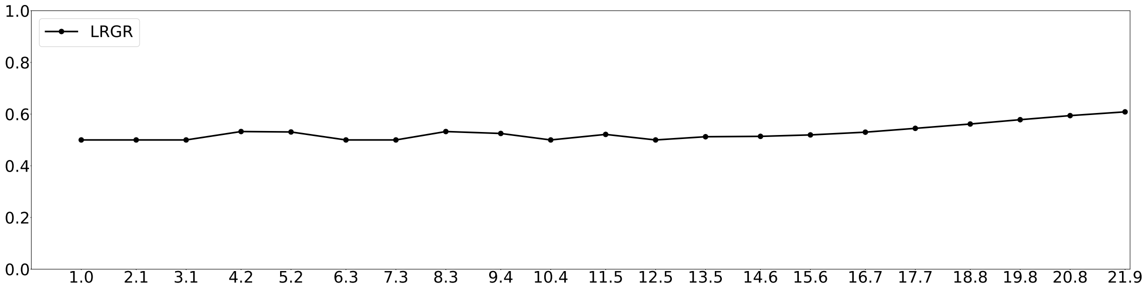

Figure 39. Trajectory 3 to goal 2. The horizontal axis measures the length of the observed trajectory. The vertical axis measures the probability of goal 2.

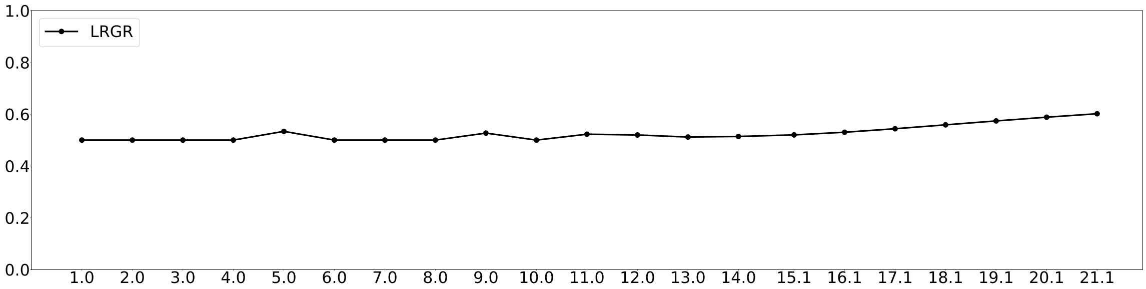

Figure 40. Trajectory 4 to goal 2. The horizontal axis measures the length of the observed trajectory. The vertical axis measures the probability of goal 2.

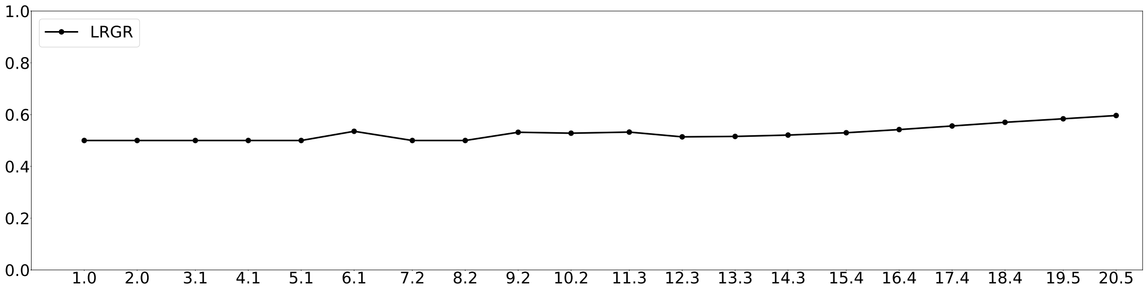

Figure 41. Trajectory 5 to goal 2. The horizontal axis measures the length of the observed trajectory. The vertical axis measures the probability of goal 2.

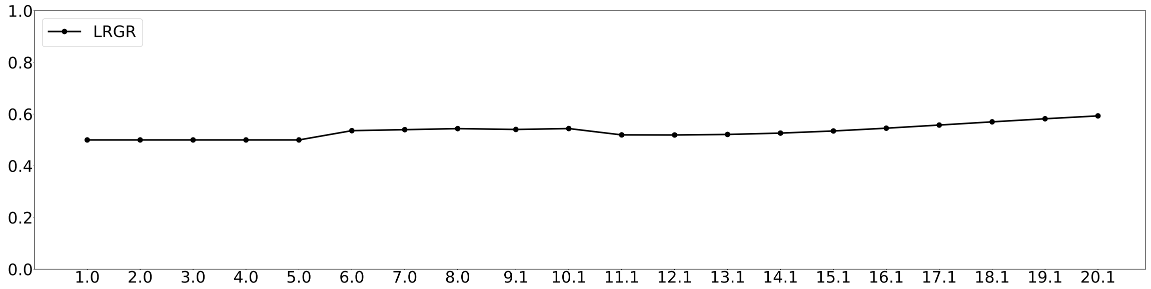

Figure 42. Trajectory 6 to goal 2. The horizontal axis measures the length of the observed trajectory. The vertical axis measures the probability of goal 2.

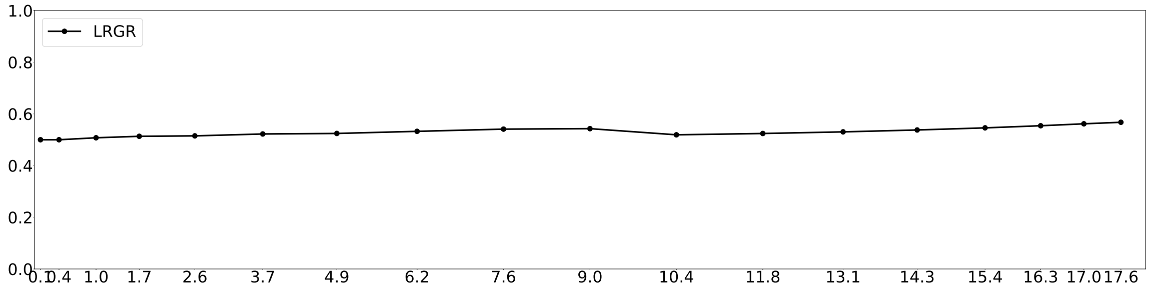

Figure 43. Trajectory 7 to goal 2. The horizontal axis measures the length of the observed trajectory. The vertical axis measures the probability of goal 2.

APPENDIX C. MEAN AND STANDARD DEVIATION OF NORMALIZED REACTION TIME OF ACCURATE RESPONSES

The experiment carried out by Berkovitz and Parush is described in [7]. To make this report self-contained, we also include here the summarized data used for comparing human response times with the algorithm responses.

We contacted Berkovitz and Parush, and are grateful that they shared the raw data with us, directly. From the data provided, we computed the *normalized* reaction time of each subject, for each trajectory, by dividing the measured reaction time, by the time necessary for the robotic arm to complete movement along the trajectory, beginning to end. Incorrect responses by subjects were filtered out. Then we took the mean normalized reaction time of each trajectory, over all subjects.

Table 1 below provides the computed mean and standard deviations of the normalized reaction times of human subjects (with correct responses). The reported means are the values used in the experiments reported above.

Goal 1	Mean	Std.
Trajectory 1	0.454252379	0.136958069
Trajectory 2	0.475543749	0.166887203
Trajectory 3	0.456414554	0.130865151
Trajectory 4	0.424790616	0.157788206
Trajectory 5	0.40941653	0.132020057
Trajectory 6	0.388984193	0.131703435
Trajectory 7	0.321730548	0.131877022

Goal 2	Mean	Std.
Trajectory 1	0.476452655	0.12710335
Trajectory 2	0.494634221	0.099861754
Trajectory 3	0.504947053	0.098553462
Trajectory 4	0.460734438	0.119508786
Trajectory 5	0.438795139	0.152004831
Trajectory 6	0.441575006	0.093543141
Trajectory 7	0.411734009	-

Table 1. Mean and standard deviation of normalized reaction times of human subjects with correct responses, as used in the experiments reported above. Values were computed from data provided by Berkovitz and Parush.
